# High mobility Si_0.15_Ge_0.85_ growth by using the molten target sputtering (MTS) within heteroepitaxy framework

**DOI:** 10.1038/s41598-019-47723-2

**Published:** 2019-08-09

**Authors:** Hyun Jung Kim

**Affiliations:** National Institute of Aerospace (NIA), 100 Exploration way, Hampton, VA 23666 USA

**Keywords:** Materials science, Engineering

## Abstract

High-speed SiGe film is promising use in photonics and electronics technologies continue to replace Si-based devices. High mobility Si_0.15_ Ge_0.85_ film on sapphire was grown at 890 °C substrate temperature by using a conventional magnetron sputtering system within the heteroepitaxy framework. 890 °C substrate temperate is impractical for commercial device manufacturing due to long thermal soak, loading time, and costly process. To leverage the practical SiGe device applications, the Molten Target Sputtering (MTS) techniques is developed. The MTS is an economic and robust process from high flux density and liquid-state of molecules benefits. At 500 °C, the lowest substrate temperature, high mobility Si_0.15_Ge_0.85_ film with continues morphology and 99.7% majority-orientation were grown by using the MTS. The hall electron mobilities of the Si_0.15_Ge_0.85_ grown at 500 °C are 456 cm^2^V^−1^s^−1^ and 123.9 cm^2^V^−1^s^−1^ at 5.59 × 10^18^ cm^3^ and 3.5 × 10^20^ cm^3^ carrier concentration at 22.38 °C, respectively. The values are 550% higher hall electron mobilities than that of Si at equivalent carrier concentration and temperatures. We envision that the MTS is beneficial for the heteroepitaxy framework film growth that requires high substrate temperature to overcome the large lattice parameter mismatch between film and substrate.

## Introduction

The demand for high-tech devices such as smart phones, tablet PCs, security systems, and other advanced electronic hardware relies on high-speed processors for computing. The performance of traditional silicon chips scales by fitting more and more transistors into a constant area or volume of silicon (Si). However, there is a limit to the amount of usable structure that can be incorporated into a Si wafer^1^. Therefore, a new paradigm is needed to continue to improve the performance of solid-state electronic devices. One promising concept for next generation CPUs is to use silicon-germanium (Si_1−x_Ge_x_, SiGe) alloys because electrons travel over 100 times faster in SiGe than in pure Si from the low effective masses associated with Ge^2^. Vastly superior electron mobility of Si_1−x_Ge_x_ on a dielectric substrate is promising use in photonics technologies continue to replace Si-based solid-state electronic devices. Although SiGe can theoretically provide semiconductor devices with better performance than Si-based devices, fabricating high quality SiGe on dielectric substrate is technically challenging due to the lattice constant mismatching between the SiGe and substrate^3–5^.

My team at the NIA/NASA reported the super-heteroepitaxy growth technology for single crystal growth of cubic or zinc-blend structure SiGe on trigonal sapphire substrate^6–9^. Within the super-heteroepitaxial framework, 850 °C substrate temperature and 200 W magnetron gun power on a conventional magnetron sputtering are required for 99% single crystal Si_0.15_Ge_0.85_ growth on *c*-plane sapphire substrate and high mobility^6–9^. The results show that the crystallinity and surface morphology are heavily dependent on the substrate temperature and magnetron gun power, improving with an increase in the parameters (Supporting Information 1). 850 °C is the temperature as read from a thermocouple attached to a holder behind the substrate. The actual substrate temperature is less than the thermocouple reading temperature because of the heat gradient in the sample holder and the infrared light passing through the transparent sapphire substrate. Recently, a new model showed that both pure Ge and Si_1−x_Ge_x_ can form 98% single crystal films at 600 W gun power and the 450–550 °C substrate temperature based on thermal expansion calculations^10^. Despite the promising model, the film growths at the 500 °C have produce a mix of terraces and droplets, which is not qualified for high-speed device fabrication.

The high substrate temperature is impractical for commercial device application as is a costly process from long thermal soak times and often not repeatable in quality to produce due to a difficult of a thermally uniform wafer^7,11–13^. Low temperature process for a single crystal Si_0.15_Ge_0.85_ film growth is feasible when a thin film deposition system could melt the Ge target before reach to the high substrate temperate and provides a high deposition rate of molecules than the convention magnetron sputtering. To leverage the practical capabilities, simple design of new technique for Si_0.15_Ge_0.85_ film growth with decreased thermal loading is presented and named Molten Target Sputtering (MTS)^14,15^.

The MTS has a thermally isolate magnetron gun between the target and water cooling plate in the conventional magnetron sputtering system. The thermally isolated magnetron gun melts the Ge target before the ejected molecules from the magnetron gun reach to the substrate. The molten Ge target of the MTS provides a high flux density of molecules similar to the thermal evaporation for Si_1−x_Ge_x_ film growth. For the first time in the literature, the continuous morphology and single crystal Si_0.15_Ge_0.85_ films with 99.7% majority-orientation were grown at 500 °C, the lowest substrate temperature, within the super-heteroepitaxy framework by using the MTS. We envision that the MTS is beneficial for the heteroepitaxy framework film growth that requires high substrate temperature to overcome the large lattice parameter mismatch between film and the substrate.

## Molten Target Sputtering (MTS) Description

The benefits of hot target sputtering from an evaporation factor in productivity and film quality were reported from 2009^16–18^. The idea was extended to the High Power Impulse Magnetron Sputtering (HPIMS) for an additional energy and flux tuning of a sputtered material towards the substrate. Even concerning the ideas of melting the target material have been reported, these systems requires additional apparatus such as a power unit and beam source to enhance the plasma temperature^19^.

**Figure 1:**
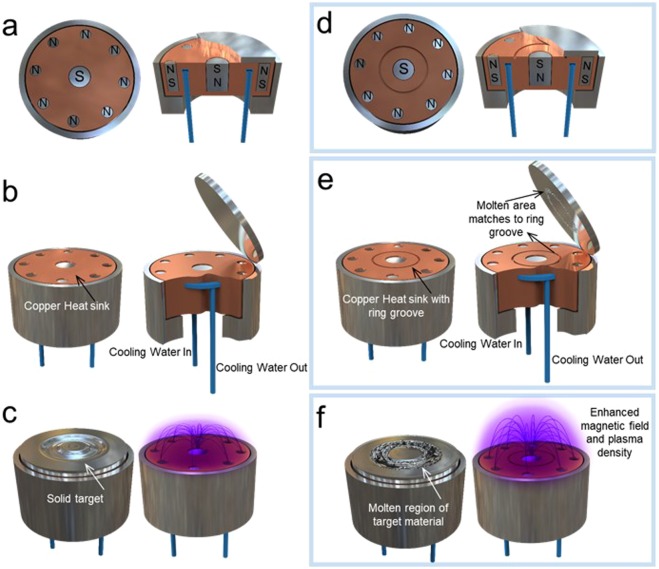
Top and side view schematics of conventional magnetron sputtering and modified magnetron Molten Target Sputtering (MTS) guns. (**a**–**c**) conventional magnetron sputtering gun: top- and side-view with magnets (**a**), turbulent cold water circulation to cool the magnetron electrode (**b**), and its magnetic field configuration and plasma (**c**). (**d–f**) Molten target sputtering gun: top- and side-view with ring groove with magnets (**d**), turbulent cold water circulation to cool the magnetron electrode but partial heating of the target material for melting (**e**), and its magnetic field configuration and plasma from the molten region of target material (**f**).

The newly developed MTS is the system with simple modification of a magnetron gun to melt target for high quality film growth without any additional apparatus. The modified magnetron gun isolates the target thermally from water cooling for melting. In conventional magnetron sputtering, a copper heat sink equipped with internal water cooling channels sits underneath the target, cooling the entire target volume, as shown in Fig. 1a–c. The MTS method (Fig. 1d–f) involves removing a small ring of material ~1–2 mm deep and wide from the copper heat sink^14,15^. The volume of target material over this ring experiences reduced conductive cooling and increased heat since the conduction passage of the thermal energy to a water cooling channel removed by the ring groove below the target. Accordingly, the toroidal area at the target surface is heated and melted. The magnetic arrangement, where one pole is positioned at the central axis of the target and the opposite pole is circumferentially placed around the outer edge of the target, confines the electrons along the magnetic flux lines near the target and keep the Gaussian-shape plasma plume^20^ (Supporting Information 2). As-received flat target is vulnerable to liquid-state molten material rolling off, so etching trench on the target comes with the MTS process or recycle a target with the plasma trace after the conventional sputtering process before the molten process (Supporting Information 3).

The MTS method provides high flux density and liquid-state of molecules from the modified magnetron sputtering gun without any additional apparatus, which is exhibited in both evaporation and magnetron sputtering (Supporting Information 4 and 5). The evaporation-involved sputtering from the MTS enables enhancement of power density of target and the flux density of the molecules. The flux density of the molecules from the MTS is similar or higher than the hot target sputtering target. The power density was demonstrated in SiZrO and Ge, where the rates are ≤55 W/cm^2^ for cold targets, 55~75 W/cm^2^ for hot targets, and ≥75 W/cm^2^ for molten targets^21–23^. The MTS supports an extra substrate heating because there would be significant thermal load from the 15 cm distance between the molten target and the topmost substrate surface. From the MTS benefits of the molten Ge before reach to the substrate and high flux density of molecules, low temperature process for single crystal Si_0.15_Ge_0.85_ growth with high mobility is feasible. We choose SiGe because both high substrate temperature and high flux density of molecules are the critical parameters for high quality and single crystal film growth (Supporting Information 1).

## SiGe Film Growth by Using the MTS

**Figure 2:**
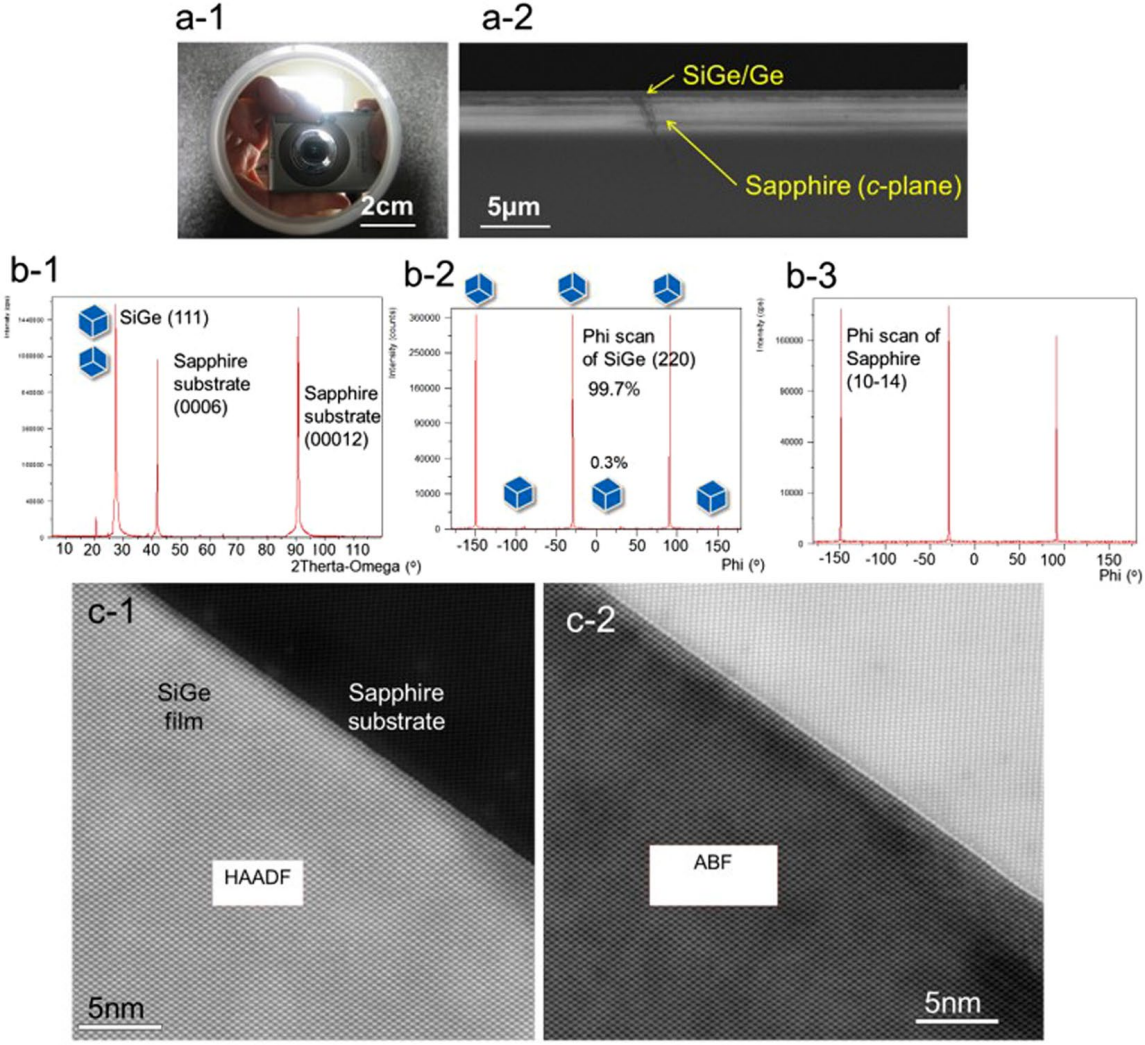
Crystallinity of SiGe on sapphire (Al_2_O_3_) film grown by molten target sputtering. Photo of SiGe with mirror-like surface (**a-1**) and cross-section of SEM image of the same sample (**a-2**). Symmetric *θ*–*2θ* XRD scan shows only the SiGe (111) reflection at 2*θ* ~27.6 degrees along with the Al_2_O_3_ (003), (006), (009), and (0012) reflections (**b-1**). SiGe (220) scan shows three strong reflections (offset by 120 degrees) with three small reflections rotated 60 degrees from them indicating greater than 99.7% in-plane orientation of the SiGe (**b-2**), and sapphire ($10\bar{1}4$) scan show three strong reflections (**a-3**). HAADF (High-Angle Annular Dark-Field) image of the SiGe and sapphire interface (**c-1**). ABF (Annular Bright Field) image of the SiGe and sapphire interface (**c-2**).

In order to demonstrate the advantages of the MTS technique, single crystal Si_0.15_Ge_0.85_ films were grown at 500 °C substrate temperature on *c*-plane sapphire substrates. Based on the substrate temperature calibration result, the actual substrate temperature is 32 °C less than the thermocouple reading at 500 °C. This is considerably lower than the previous growth temperature of ~850 °C^6,7,24^. The 500 °C substrate temperature was chosen for Si_0.15_Ge_0.85_ growth based on the thermal expansion model^10^. The SiGe film shown in Fig. 2 resulted from MTS grown at 500 °C.

Figure 2(a-1) shows the photo of wafer with a mirror-like surface. The SiGe on Al_2_O_3_ was a continuous smooth film as shown in Fig. 2(a-2). X-Ray diffraction (XRD) data of these highly single crystalline (>99.7%) SiGe layers on *c*-plane sapphire wafers are shown in Fig. 2(b). A symmetric *θ*–2*θ* scan, which probed the surface normal direction of the sample, showed only the SiGe (111) reflection at 2*θ* ~27.5 degrees with high intensity (1650000 cps) and narrow full-width at half-maximum (0.018 FWHM) along with the Al_2_O_3_ (003), (006), (009), and (0012) reflections. No other SiGe reflections, such as the (220), (311), or (400), appeared in the symmetric scan, verifying the (111) plane matched to the sapphire *c*-plane. This indicates a high-quality, uniform film along the wafer axial direction. The in-plane orientation of the crystallites was measured with a scan of the SiGe (220) reflections. Three strong reflections, offset by 120 degrees in the scan, indicated the majority in-plane orientation of the SiGe with three small peaks indicating the 60 degrees rotated (twinned) minority in-plane orientation. The majority percentage of the SiGe crystallites was determined by comparing the integrated areas under each peak, reaching a maximum of 99.7%. 4 k × 4 k STEM images (Fig. 2(c)) provide a field of view with high-resolution detail of no twin and highly crystalline SiGe film grown on *c*-plane sapphire. The epitaxial relationship between the SiGe film and the sapphire structure is (111) SiGe//(0001) Al_2_O_3_ and [011] SiGe // [$01\bar{1}0$] Al_2_O_3_.

**Figure 3:**
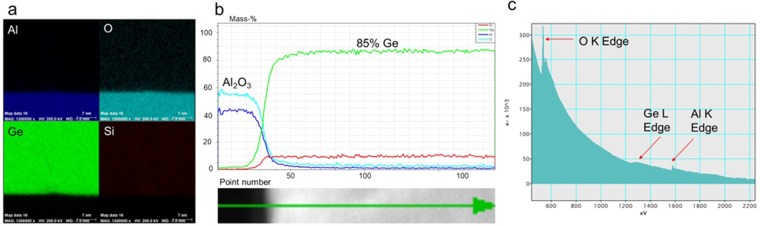
Quantitative STEM-EDX mapping (**a**) and line scan of EDX mapping of SiGe film (**b**). Si and Ge uniformly distributed in the film and 85% of Ge concentration confirmed by EDX line profile of the film. Total EELS signal of SiGe film (**c**) Ge L edge is visible but the Si K edge is not visible from the total EELS signal due to fast acquisition and relatively lower concentration of Si.

Figure 3(a,b) show the TEM mapping and quantitative line scan of STEM- Energy Dispersive X-ray (EDX) mapping. The mapping shows the Si, Ge, Al, and O elements and homogenous SiGe with 85% of Ge concentration. The total Electron Energy Loss Spectroscopy (EELS) signal of the SiGe film shows no Si K edge due to fast acquisition and relatively lower concentration of Si (c).

**Figure 4:**
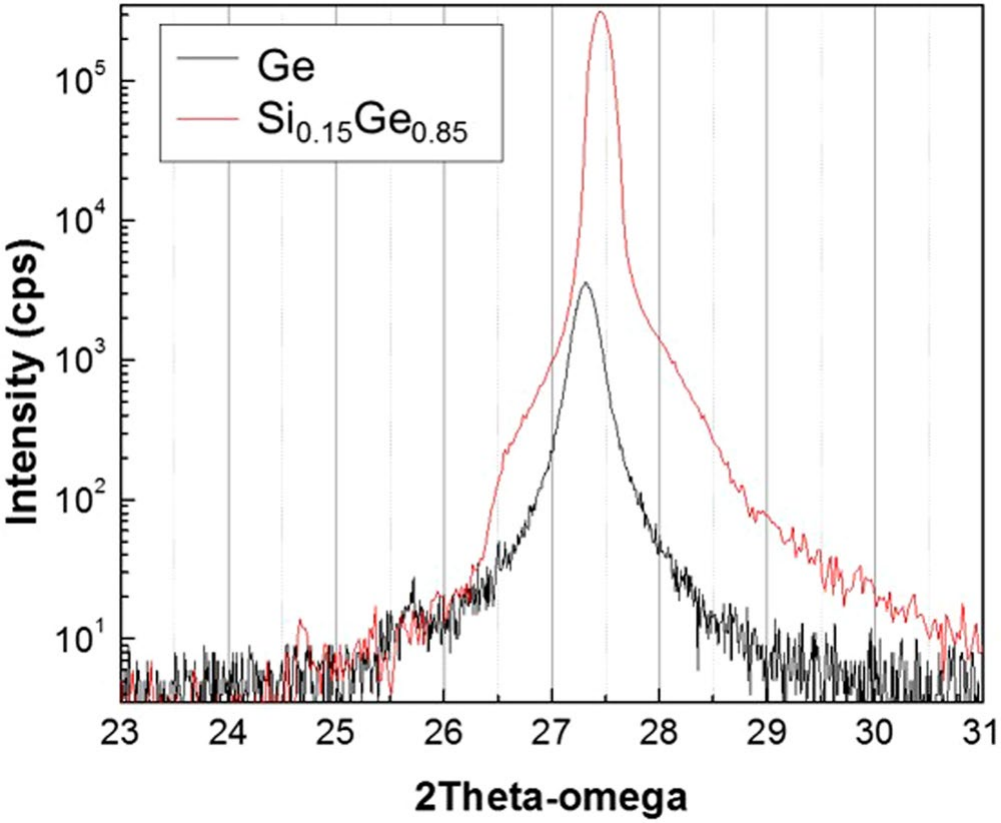
The (111) diffraction peaks from XRD *2θ-Ω* scan. The (111) Si_1−x_Ge_x_ and Ge peaks locate at 27.48° (red) and 27.25° (black), respectively. It shows the composition of the Si_1−x_Ge_x_ film is Si_0.15_Ge_0.85_.

Figure 4 shows X-ray diffraction (XRD) *2θ-Ω* scan of Si_1−x_Ge_x_ (red line) and Ge (black line) grown on the *c*-plane sapphire substrate. By increasing Ge content, the (111) diffraction peak in Si_1−x_Ge_x_ progressively shifts to lower angles from that of Si in comparison with Ge. The variation in lattice constant follows the general trend of bulk Si_1−x_Ge_x_ in Vegard limit^25,26^. The XRD result confirms the high Ge-content of Si_1−x_Ge_x_ as the composition of Si_0.15_Ge_0.85_.

**Figure 5:**
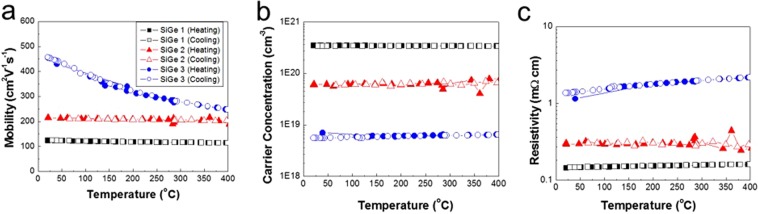
(**a**) Hall electron mobility (µ) as a function of temperature (both heating and cooling) for three samples with different amount of boron (B) doping (denoted SiGe 1, SiGe 2, and SiGe 3). (**b**) Carrier concentration (*n*) as a function of temperature for the same three samples. (**c**) Resistivity of the same three samples as a function of temperature.

Figure 5 shows the Hall mobilities, carrier concentrations, and resistivities as a function of temperature, both heating and cooling, and boron doping concentrations. The highest mobility, 456 cm^2^V^−1^s^−1^ at 22.38 °C occurs for a carrier concentration of 5.59 × 10^18^ cm^−3^. This mobility is about 550% higher than that of the Si given by Masetti *et al*. and similar to the Ge value at equivalent carrier concentrations in Fig. 5(a) ^27–29^. Both hole mobility and resistivity of (1) 3.5 × 10^20^ cm^3^ carrier concentrations are 123.9 cm^2^V^−1^s^−1^ and 0.146 mΩcm, (2) 6.11 × 10^19^ cm^3^ carrier concentrations are 214.4 cm^2^V^−1^s^−1^ and 0.296 mΩcm, (3) 5.59 × 10^18^ cm^3^ carrier concentrations are 456 cm^2^V^−1^s^−1^ and 1.36 mΩ·cm at 20 °C, respectively as summarized in Table 1. 5.59 × 10^18^ cm carrier concentration SiGe film exhibits a gradual decrease by increasing temperature but not the two other samples with higher carrier concentration. This implies that carrier mobility is independent of temperature in the heavily impurity-doped SiGe with such relatively high Ge contents^30^. Figure 5(b) shows the carrier concentrations as a function of temperature for the SiGe films. The slight increase in carrier concentration up to 400 °C has been observed for 6.11 × 10^19^ cm^3^ boron-doped SiGe samples only. The carrier concentrations of the low and high impurity-doped crystals are almost independent of temperature up to 400 °C.

**Table 1:** Hole mobility and resistivity based on the carrier concentration at 20 °C.

Si/Ge ratio of Si_x_ Ge_1−x_	Film deposition temperature (°C)	Carrier concentration (cm^2^V^−1^s^−1^)	Hole mobility (cm^3^)	Resistivity (mΩcm)	Temperature for measurement (°C)
Si_0.15_Ge_0.85_	500	3.5 × 10^20^	123.9	0.146	20
Si_0.15_Ge_0.85_	500	6.11 × 10^19^	214.4	0.296	20
Si_0.15_Ge_0.85_	500	5.59 × 10^18^	456	1.36	20

The higher mobiNlity of Si_0.15_Ge_0.85_ grown by MTS compare to the Si indicates monocrystalline film and continuous morphology.

## Discussions

The high flux density of molecules and enhancement of power density of the MTS results in a high deposition rate over conventional magnetron sputtering. There are three potential mechanisms for the enhanced deposition rate: particle-induced erosion^31^, sputtering yield increase^32^, and target evaporation (or sublimation).

First, the vapor pressure change in front of the target from the MTS enables enhancement of the flux density of molecules. As the Ge target is molten during the MTS, the target temperature is clearly above its melting point of 938.2 °C (1211.35 K). The vapor pressure depends on temperature and can be estimated from the vapor pressure curve measured by the Knudsen effusion method. At the equilibrium temperature of the molten Ge target, 1236.85 °C (1510 K), the Ge vapor pressure is 1.35 × 10^−5^ atm. and the pressure values is increased as the temperature increases^33^. Dmitrii Sidelev *et al*.^34^ presents a comparison of magnetron sputtering with cooled and hot chromium (Cr) targets using the properties of a Cr deposited film. Based on these results, the main mechanism for the high flux density of molecules is target evaporation where the particle-induced erosion and the sputtering yield increase slightly. Secondly, MTS increases the flux density of the molecules similar to or higher than the hot target sputtering target. It has been revealed that sublimation and local evaporation are the most important mechanisms of erosive flux increase in hot target sputtering^17,35,36^. The evaporation controls the deposition rate of the molten target the same as in hot target sputtering and the flux density is even higher as the erosive flux density increases exponentially dependent on the target temperature^31,37^. Moreover, the weaker binding of the liquid-state atoms on the molten target compared to solid-state targets leads to easier ejection, thereby increasing the flux density of molecules^38^. Even when the reasons for the high flux density of molecules from the MTS are addressed, further evaluation of the plasma properties, local ionization, and conferment would be interesting when the evaporation occurs simultaneously with the sputtering.

One of the explanations of the 500 °C growth mechanism by using the MTS from the high flux density of molecules is based on the surface kinetic model^7^. The surface kinetic model proposed by Park *et al*. explains the Si_0.15_Ge_0.85_ heteroepitaxy one twin dominates over the other due to differences in diffusion barrier energies. The major twin contributes to the single crystal growth rests on sites with deep energy wells, while minor twins start on atomic sites with much smaller energy wells. The deeper major twin can be “quickly fill and frozen” in place before diffusion move them from the high flux density and high power density benefits of MTS. Moreover, adatoms will diffuse out of minor twin sites into major twin sites from the benefit of the molten Ge as the surface adatoms increase the mobility. Finally, the single crystal and continuous morphology Si_0.15_Ge_0.85_ was grown at 500 °C by using the MTS from the high deposition rate and molten Ge target benefits.

## Conclusions

100 times faster electron mobility of Si_1−x_Ge_x_ is promising use in photonics technologies continue to replace Si-based solid-state electronic devices. For the high-speed device fabrication, the Si_1−x_Ge_x_ film must be a monocrystalline, a continuous and uniform morphology, and high Ge-contents.

Over 850 °C substrate temperature on a conventional magnetron sputtering is required for 99% single crystal and continuous morphology Si_0.15_Ge_0.85_ thin film on *c*-plane sapphire substrate. The high substrate temperature is impractical for commercial device application due to a costly process from long thermal soak times and often not repeatable in quality to produce as a difficulty of a thermally uniform wafer. To leverage the practical capabilities, we use a Molten Target Sputtering (MTS) which has modified magnetron gun which can melt a sputtering target by minimizing the conductive heat sink attached to the conventional magnetron sputtering system. The MTS provides benefits of high flux density and liquid-state of molecules, which is exhibited in evaporation and magnetron sputtering.

We have demonstrated the first in literature, continuous morphology and single crystal Si_0.15_Ge_0.85_ films with 99.7% majority-orientation at 500 °C substrate (44% reduction compare to 850 °C) temperature from the benefits of the MTS. The hall electron mobilities of the Si_0.15_Ge_0.85_ are 456 cm^2^V^−1^s^−1^ and 123.9 cm^2^V^−1^s^−1^ at 5.59 × 10^18^ cm^3^ and 3.5 × 10^20^ cm^3^ carrier concentration at 22.38 °C, respectively. The values are 550% higher hall electron mobilities than that of Si at equivalent carrier concentration and temperatures.

The growth mechanism of the Si_0.15_Ge_0.85_ at 500 °C by using the MTS can be explained by the surface kinetic model. An interesting future study would grow a single crystal Si_0.15_Ge_0.85_ below 500 °C or even at room temperature with MTS. We envision that the MTS is beneficial for the heteroepitaxy framework film growth that requires high substrate temperature to overcome the large lattice parameter mismatch between film and the substrate.

## Methods

### Sample preparation and film deposition

A 2-inch *c*-plane sapphire substrate (1-side polished, MTI Corporation) was cleaned to obtain a surface without native oxide layer or organic and inorganic contaminants, such as the chemical mechanical polishing (CMP) slurry by using acetone (ACS grade), iso-propanol (ACS grade), and deionized water sequentially before deposition. The cleaned sapphire wafer was loaded on a molybdenum wafer holder with the epi-ready side facing downward in the bottom-target design sputtering chamber. The wafer was preheated by using a PBN/PG (Pyrolytic Graphite) ceramic heater (Momentive Inc.) through two-steps (200 °C and 1000 °C) at the base pressure of the sputtering chamber, 1 × 10^−6^ Torr. The sapphire wafer was baked at 200 °C for 20 minutes to remove water vapor, any volatile contaminants, and native oxide layer. After this, the wafer was heated to 1000 °C for another 60 minutes for clean and single termination of the sapphire surface^6,39,40^. To prevent thermal shock and wafer cracking, the substrate ramped to 1000 °C with the increment of 5 °C/minute. The actual substrate temperature is measured by using a 2-inch (50.8 mm) diameter thermocouple instrumented wafer (Prime technology Co, LTD, PW02 sensor, K-type, 5-point reading) and a data monitor (GraphTec Inc, GL820 multi-channel logger) at a chamber pressure of 2.6 × 10^−6^ Torr with the main shutter closed. The equation of the temperature calibration shows *y* = 0.9559*x* − 9.557, where *x* is the set temperature and *y* the actual substrate temperature. The pressure for the SiGe film deposition was 7mTorr with a 5 sccm of high-purity, research grade Ar (99.9999%) flow. The Si (undoped, 99.999%, Kurt J. Lesker) target-loaded magnetron gun ran at 200 W. 200 W magnetron gun power also was applied to a used Ge (n-type, 5–40 Ω·cm, 99.9999%, Kurt J. Lesker) target which has 0.05 inch (1.27 mm) depth trace mounted onto the modified sputtering gun. The Ge target was melted during the MTS process. The Si target shutter opened for 5 minutes for the pre-sputtering of the initial Si-rich monolayers of SiGe and was followed by the opening of the Ge shutter. For the mobility test, the boron target with 1~3 W RF power was co-sputtered during the SiGe film deposition process, which resulted in heavy doping above 10^19^/cm^3^. During the 30 minutes deposition, the growth rates of both Si and Ge were monitored from the quartz thickness monitor (Maxtek, Inc.). The deposition rates of the Ge films on the rotating substrate holder (10 rpm) were 4.44 Å/second (deposition rate = 0.0215 × power + 0.14) and 28.4 Å/second for cooled and molten target, respectively. The deposition rate from the molten Ge target was approximately 6.4 times higher than in cold target.

### Photography and SEM characterization

The photography images were taken by using a Canon Powershot SD400 5MP Digital Elph Camera. The SEM cross-section of SiGe on sapphire was taken from the SEM (JSM-5800).

### XRD characterization

The SiGe film was scanned with a Panalytical X’Pert Pro XRD system along the 2θ-ω and phi (*Φ*) scan. The vertical atomic alignment was measured with a symmetric *2θ-*ω scan, which probes the surface normal direction from 5 to 120^o^ to establish overall film quality. The majority percentage of the SiGe crystallites was determined by comparing the integrated areas under each peak. The *Φ* scan is parallel with the (111) SiGe surface normal, and each scan completed one whole revolution of the wafer at a constant position designed to find all the {220} reflections. A similar *Φ* scan is done with the sapphire (0001), parallel with the (111) SiGe surface, to find all the $\{10\bar{1}4\}$ reflections^8,9^. A high quality SiGe film will have three peaks in the SiGe scan, corresponding to the three {220} planes in the film. The presence of six peaks, 60° offset from one another, corresponds to two twins, rotated 60° with respect to one another, each with three {220} reflections. Relative peak heights of these twins are compared to determine how much of each twin exists in the SiGe film.

### TEM sampling and TEM characterization

TEM samples were prepared using a FEI Quanta 3D FEG focused ion beam (FIB)^41^. The interface between SiGe and Al_2_O_3_ was characterized using an FEI Titan 200 kV TEM. An aberration-corrected scanning transmission electron microscope (STEM) equipped with a high-angle annular dark field (HAADF) detector and an annular bright field (ABF) detector, and an energy-dispersive X-ray spectroscopy (EDX) were used to analyze the atomic structure of SiGe specimen. All the TEM images and EDX mapping images were collected using a 200 kV acceleration voltage. The STEM image was obtained with an 18 mrad convergence angle using 50 µm C2 aperture. The HAADF STEM image was developed using Fischione HAADF and FEI DF4 detectors. The ABF STEM image was obtained using a combination of FEI DF4/DF2 detectors after adjusting the suitable camera length. The EDX was used to detect the chemical element distribution of the SiGe/Al_2_O_3_ interface. In this method, a coherent focused probe scanned across the specimen, and the resultant x-ray emission spectrum was recorded at each probe position. These spectra were then used to construct an elemental line scan. The acquisition time for a <112> zone-axis of SiGe on sapphire EDX mapping was 11 minutes under a 512 × 512 pixel image size, 250pA beam current, and spot size of 6. The Electron Energy Loss Spectroscopy (EELS) has been performed over a range of 400 ~ 2300 eV.

### Hall-effect measurements

The Hall mobility, carrier concentration, and resistivity were measured as a function of temperature from room temperature, 20 °C to 400 °C using a Physical Property Measurement System (Quantum Design) at the NASA-JPL thermoelectric laboratory^42,43^. A square shaped van der Pauw geometry sample (10 mm × 10 mm), with ohmic contact (<1 mm × 1 mm) at the sample corners, was used for the measurement, under a reversible 1 Tesla magnetic field.

## Supplementary information

High mobility Si<sub>0.15</sub>Ge<sub>0.85</sub> growth by using the molten target sputtering (MTS) within heteroepitaxy framework

## References

[1] Keyes RW (2005). Physical limits of silicon transistors and circuits. Rep. Prog. Phys..

[2] Thwaites MJ, Reehal HS (1997). Growth of single-crystal Si, Ge, and SiGe layers using plasma-assisted CVD. Thin Solid Films.

[3] Kim, H. J., Park, Y., Bae, H. B. & Choi, S. H. High-electron-mobility SiGe on sapphire substrate for fast chipsets, *Advances in Condensed Matter Physics*, **785415** (2015).

[4] Gomez L, Ni Chleirigh C, Hashemi P, Hoyt L (2010). Enhanced hole mobility in high Ge content asymmetrically strained-SiGe p-MOSFETs. IEEE Electron Device Letters.

[5] O’Regan T, Fischetti M (2007). Electron mobility in silicon and germanium inversion layers: The role of remote phonon scattering. J. Comput. Electron..

[6] Kim HJ, Duzik A, Choi SH (2018). Lattice-alignment mechanism of SiGe on Sapphire. Acta Materialia.

[7] Park Y, King C, Choi S (2008). Rhombohedral epitaxy of cubic SiGe on trigonal c-plane sapphire. J. Cryst. Growth..

[8] Kim HJ, Bae H, Park Y, Lee K, Choi S (2012). Temperature dependence of crystalline SiGe growth on sapphire (0001) substrates by sputtering. J. Crystal Growth.

[9] Park, Y., Choi, S., King, G. & Elliott, J. R. U.S. Patent US8,226,767, July 24 (2012).

[10] Duzik, A. J. & Choi, S. H. Low temperature rhombohedral single crystal SiGe epitaxy on c-plane sapphire. *Proc. SPIE* 9802, Nanosensors, Biosensors, and Info-Tech Sensors and Systems 98020D, 10.1117/12.2218646 (2016).

[11] Alterovitz A, Mueller H, Croke T (2004). High mobility SiGe/Si transistor structures on sapphire substrates using ion implantation. J. Vac. Sci. Technol. B.

[12] Ismail K, Arafa M, Saenger L, Chu O, Meyerson S (1995). Extremely high electron mobility in Si/SiGe modulation‐doped heterostructures. Appl. Phys. Lett..

[13] Koester J (2001). SiGe pMODFETs on silicon-on-sapphire substrates with 116 GHz f_max_. IEEE Electron Device Lett..

[14] Kim, H. J. & Choi, S. H. Molten target sputtering (MTS) deposition for enhanced kinetic energy and flux of ioized atoms. US Application (US20170268122A1) (2018).

[15] Kim, H. J. & Choi, S. H. Rhombohedron epitaxial growth with molten target sputtering. US Application (US 20170145589A1) (2018).

[16] Vicek J, Zustin B, Rezek J, Burcalova K, Tesar J (2009). Pulsed magnetron sputtering of metallic films using a hot target. Proc. of the annual technical conference-society of vacuum coaters..

[17] Bleykher GA, Borduleva AO, Krivobokov VP, Sidelev DV (2016). Evaporation factor in productivity increase of hot target magnetron sputtering systems. Vacuum..

[18] Sidelve DV (2018). Hot target magnetron sputtering for ferromagnetic films deposition. Surface & Coatings Technology.

[19] Lundin D, Sarakinos K (2012). An introduction to thin film processing using high-power impulse magnetron sputtering. J. of Mat. Res..

[20] Kelly PJ, Arnell RD (2000). Magnetron sputtering: a review of recent developments and applications. Vacuum..

[21] Musil J, Satava V, Baroch P (2010). High-rate reactive deposition of transparent SiO_2_ films containing low amount of Zr from molten magnetron target. Thin Solid Films.

[22] Taga Y, Takahasi P (1997). Role of kinetic energy of sputtered particles in thin film formation. Surface Science.

[23] Yuryeva AV, Shabunin AS, Korzhenko DV, Korneva OS, Nikolaev MV (2017). Effect of material of the crucible on operation of magnetron sputtering system with liquid-phase target. Vacuum.

[24] Muller K-H (1987). Role of incident kinetic energy of adatoms in thin film growth. Surface Science.

[25] Yang J, Jin C, Kim C, Jo M (2006). Band-Gap Modulation in Single-Crystalline Si_1−x_Ge_x_ Nanowires. Nano Letter.

[26] Dismukes JP, Ekstrom L, Steigmeier EF, Kudman I, Beers DS (1964). Thermal and Electrical Properties of Heavily Doped Ge‐Si Alloys up to 1300 °K. J. Appl. Phys..

[27] Jacoboni C, Canali C, Otiaviani G, Quaranta AA (1977). A review of some charge transport properties of silicon. Solid State Physics.

[28] Golikova O, Moizhes BY, Stil’bans LS (1962). ole mobility of germanium as a function of concentration and temperature. Sov. Physics Solid State.

[29] Masetti, G., Severi, M. & Solmi, S. Modeling of carrier mobility against carrier concentration in arsenic-, phosphorus-, and boron-doped silicon, *Electron Devices IEEE*, 764–769 (1983).

[30] Yonenaga I, Li WJ, Akashi T, Ayuzawa T, Goto T (2005). Temperature dependence of electron and hole mobilities in heavily impurity-doped SiGe single crystals. J. Appl. Phys..

[31] Doerner RP, Krasheninnikov SI (2004). Particle-induced erosion of materials at elevated temperature. J. Appl. Phys..

[32] Behrisch R, Eckstein W (1993). Sputtering yield increase with target temperature for Ag. Nucl. Inst. Methods Phys. Res. B..

[33] Searcy AW (1962). The vapor pressure of germanium. The Journal of Physical Chemistry.

[34] Sidelev DV, Bleykher GA, Krivobokov VP, Koishybayeva Z (2016). High-rate magnetron sputtering with hot target. Surface and Coatings Technology.

[35] Merce D, Perry F, Billard A (2006). Hot target sputtering: A new way for high-rate deposition of stoichiometir ceramic films. Surf. and Coat. Tech..

[36] Sidelev DV (2018). Hot target magnetron sputtering for ferromagnetic films deposition. Surf. and Coat Tech..

[37] Bleykher GA, Krivobokov VP, Yurjeva AV, Sadykova I (2016). Energy and substance transfer in magnetron sputtering systems with liquid-phase target. Vacuum.

[38] Konov DA, Mosunov AS, Adamov GV, Shelyakin LB, Yusaova VE (2001). Angular dependence of sputtering for nickel in ferro and paramagnetic states. Vacuum.

[39] Wei PSP, Smith AW (2001). Structure of the (0001) surface of α-Alumina. Journal of Vacuum Science and Technology.

[40] Vermeersch M, Malengreau F, Sporken R, Caudano R (1995). The aluminum/sapphire interface formation at high temperature: an AES and LEED study. Surface Science.

[41] Kim, H. J., Choi, S., Bae, H. & Lee, T. W. Transmission Electron Microscopy (TEM) sample preparation of Si_1−x_Ge_x_ in *c*-plane sapphire substrate. NASA/TM-2012-217597 (2012).

[42] van der Pauw LJ (1958). A method of measuring specific resistivity and Hall effect of discs of arbitrary shape. Philips Res. Rep..

[43] Bierwagen O, Ive T, Van de Walle CG, Speck JS (2008). Causes of incorrect carrier-type identification in van der Pauw-Hall measurement. Appl. Phys. Lett..

