# Supplementary material for: High mobility Si0.15Ge0.85 growth by using the molten target sputtering (MTS) within heteroepitaxy framework
Source: Sci Rep. 2019 Aug 9;9:11555. doi: 10.1038/s41598-019-47723-2 (PMC6689090; doi:10.1038/s41598-019-47723-2)
Supplement: Supplementary file 1 — High mobility Si0.15Ge0.85 growth by using the molten target sputtering (MTS) within heteroepitaxy framework [file 41598_2019_47723_MOESM1_ESM.docx]

**High mobility Si_0.15_Ge_0.85_ growth by using the molten target sputtering (MTS) within heteroepitaxy framework**

Hyun Jung Kim

National Institute of Aerospace (NIA), 100 Exploration Way, Hampton, VA 23666

**Supporting information 1. Super-heteroepitaxy SiGe research at NIA / NASA**

The following pertains to the research by the author and associates at the NASA Langley Research Center (NASA LaRC) / National Institute of Aerospace (NIA) which motivated development of the Molten Target Sputtering (MTS) technique.

**(1)** *Reference: “Rhombohedral epitaxy of cubic SiGe on trigonal c-plane sapphire”, Y. Park et al, J. Crystal Growth, 310, 2014-2731 (2008)*

Highly [111]-oriented rhombohedral hetero-structure epitaxy of cubic SiGe semiconductor on trigonal *c*-plane sapphire was characterized by X-Ray Diffraction (XRD).


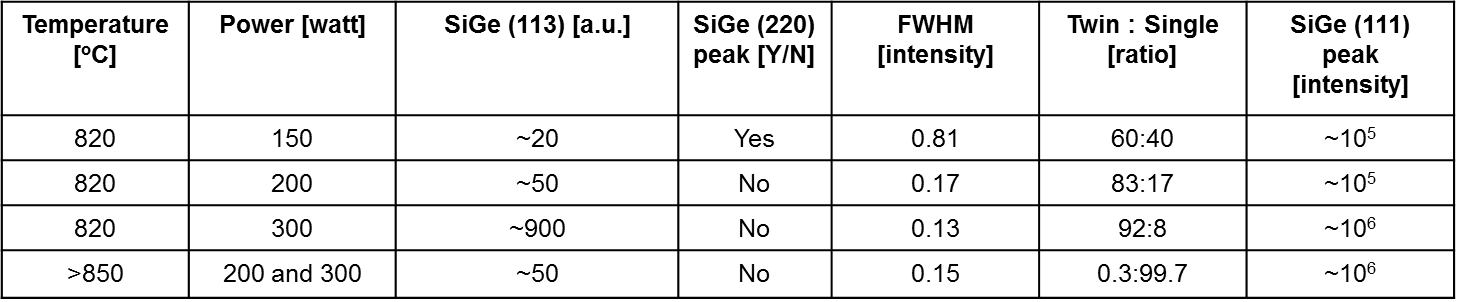


- In order to promote continuous single crystal Si_0.15_Ge_0.85_ film growth on *c*-plane sapphire substrate the substrate temperature has to exceed 850 ^o^ C substrate temperature, have 200W DC power for both Si and Ge source target, and 7sccm Ar flow with 5mTorr working pressure.

- The orientation relationship between Si_0.15_Ge_0.85_ and Al_2_O_3_ is (111)SiGe || (0001)Al_2_O_3_, [011]SiGe || [$01\bar{1}$0]Al_2_O_3_

- Both the single and twin SiGe (111) had 60^o^ rotated orientations. The type of twin formed varied between the evaluated 820^o^C and 890^o^C fabrication temperatures. The volume percentage of the twins depended on the magnetron sputtering power.

- Increasing the sputtering target power from 150W to 300W with a 820^o^C substrate temperature, the Full Width Half Maximum (FWHM) intensity was decreased, SiGe (111) peak intensity increased, and twin intensity was increased.

- By increasing the substrate temperature from 820^o^C to 850^o^C at 200W DC power, the ratio of single orientation increased, switching the ratio of the majority from twin to single

- Both the substrate temperature and Si and Ge source target power respectively determine the type of the twins and volume percentage of dominant twins.

- In order to fabricate high single dominant with low FWHM, 200-300W DC power and a substrate temperature of >850^o^C are required, as well as a smooth surface (111) Si_0.15_Ge_0.85_ film on (0001) Al_2_O_3_ (*c*-plane sapphire) substrate

**(2)** *Reference: “Temperature dependence of crystalline SiGe growth on sapphire (0001) substrates by sputtering”, H.J. Kim at al. J. Crystal Growth, 353, 124-128 (2012)*

The type of twin formed at the two temperatures, 820°C and 890°C varied. The early and final stage morphologies affected by the substrate temperatures from the Scanning Electron Microscope (SEM) and Transmission Electron Microscopy (TEM) characterizations are given below;


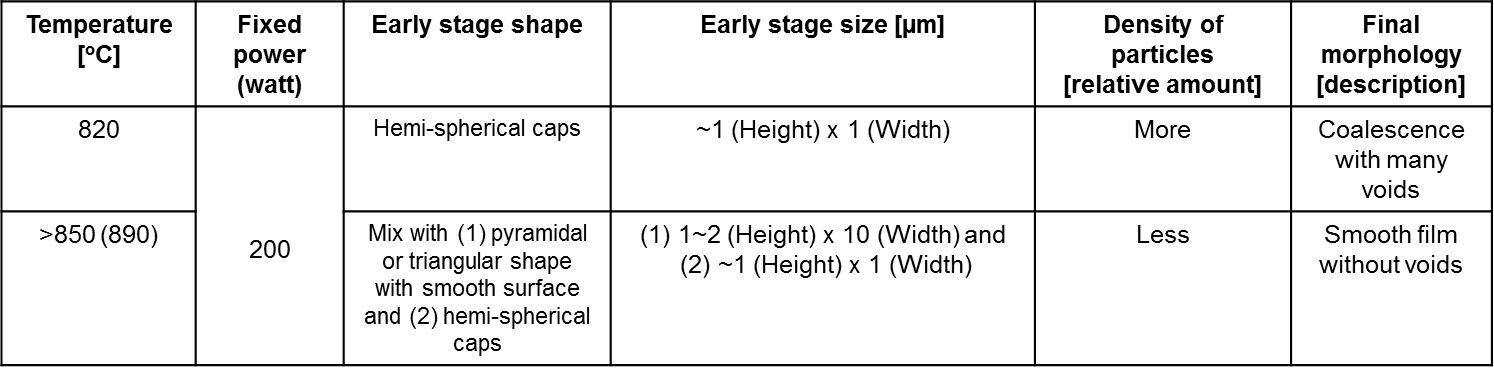


- At a substrate temperature of 820ºC, (1) early stage of film growth, each island has its own orientation. At the final stage (2) of the film growth, two non-connected islands with 60^o^ twin relationship have formed.

- With the substrate temperature higher than 850ºC, (1) early stage of the growth has two islands with 60^o^ twin relationship. A the final stage (2) of the growth, the ratio of the twins was decreased and the surface morphology became smooth without voids or noticeable boundaries

- The volume of twins was suppressed by increasing the growth temperature from 820^o^C to 890^o^C at 200W DC power. This also improved the crystalline morphology.

**(3)** *Reference: “Low temperature rhombohedral single crystal SiGe epitaxy on c-plane sapphire”, A. J. Duzik et al., SPIE proceeding volume 9802, Nanosensors, Biosensors, and Info-Tech Sensors and System, 98020D doi: 10.1117/12.2218646, (2016)*

A new model showed that both pure Ge and SiGe can form single crystal films at the 450-550°C substrate temperature based on thermal expansion calculations. Single crystal Si_0.15_Ge_0.85_ was grown at a 500°C substrate temperature and 600W DC power of the Si and Ge source target. However, the final stage film did not show a continuous film, instead forming island morphology.

**(4)** *Reference: “Lattice-alignment mechanism of SiGe on Sapphire”, Hyun Jung Kim et al., Acta Materailia, 145, 1-7 (2018)*

The paper highlights the importance of the cleanliness and a single termination of the sapphire substrate for SiGe growth within the super heteroepitaxy mechanism. The atomic layers of the unstrained Si-rich SiGe form on the O-terminated sapphire interface. After the Ge concentration increases modestly and the unstrained form of cubic structures turns into a strained structure up to the Ge concentration uniform. Finally, the lattice spacing distance of Si_0.15_Ge_0.85_ reaches normal level and the strain is relaxed.

**(5) Summary from the previous work**

| Parameters | Critical values of the parameters | Contribution of values | Value contributions for film growth | Si_0.15_Ge_0.85_ film from the critical value |
| --- | --- | --- | --- | --- |
| Magnetron gun power | 200 Watt | High deposition rate | Crystallinity | Single crystal dominated film |
| Substrate temperature | 850 °C | Ge melting | Morphology | Continuous morphology film |

The table shows the contribution of the parameters for single crystal Si0.15Ge0.85 film growth. NASA LaRC / NIA team reports the critical parameters and parameters value for uniform and continuous morphology and 99% single crystal Si_0.15_Ge_0.85_ film growth within heteroepitaxial framework. First, the substrate temperature must be higher than 850^o^C for Ge melt under working pressure and magnetron DC power must be higher than 200W for a high deposition rate from a high flux density of molecules. The magnetron gun power and substrate temperature affect the crystallinity and morphology of the film, respectively as shown in the table.

Increase the magnetron gun power represents the enhancement of power density of the target results in the high flux density of molecules. The high flux density of molecules increases the single crystal ratio in the Si_0.15_Ge_0.85_ film. 850^o^C substrate temperature is a critical value for Ge melting under the working pressure. The melted Ge on the sapphire substrate contributes the continuous film morphology.

Based on previous results, molten state along with the high deposition rate of Ge are critical parameters for uniform and continuous single crystal Si_0.15_Ge_0.85_ film growth. The previous work generated the following question: *Can we grow uniform singe crystal Si_0.15_Ge_0.85_ film at 500°C or lower substrate temperature if we melt the Ge target before it reaches the substrate with a high deposition rate? If so, which deposition system can be used to melt the target and high flux density of molecules compare to the convention sputtering?* Low temperature process for a single crystal Si_0.15_Ge_0.85_ film growth is feasible when a thin film deposition system could melt the Ge target before reach to the high substrate temperate and provides a high deposition rate of molecules.

We adapt a method, Molten Target Sputtering (MTS) which melts the Ge target before reaching to the high substrate temperature and provides a high deposition rate for Si_1-x_Ge_x_ film growth. Finally, the single crystal and continuous morphology Si_0.15_Ge_0.85_ was grown at 500^o^C by using the MTS.

**Supporting information 2. Plasma distribution of the sputtering targets**

The magnetic arrangement, where one pole is positioned at the central axis of the target and the opposite pole is circumferentially placed around the outer edge of the target, confines the electrons along the magnetic flux lines near the target and keep the Gaussian-shape plasma plume.


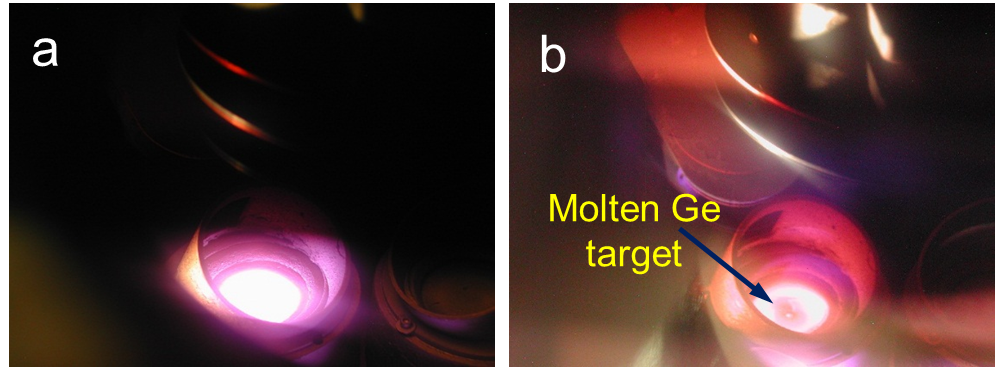


**Figure 1. Plasma differences between the conventional magnetron sputtering and the molten target sputtering.** Photo of plasma from a solid target in a conventional magnetron sputtering chamber (a). Photo of plasma from the molten target inside the sputtering chamber (b). The plasma ring around the magnet equator from the molten sputtering target (indicated by arrow) is heterogeneous versus the conventional process and the wavy zone of target is indirect evidence of the liquid state of the target.

**Supporting information 3. Target materials after two different sputtering processes**

The molten region on the target surface limits the orientation of the gun to straight up as the molten material will roll off if mounted at an angle of sideways. As-received flat target is vulnerable to liquid material rolling off, so etching trench on the molten target comes with the conventional sputtering process or recycle a target with the plasma trace after the conventional sputtering process before the molten process

**
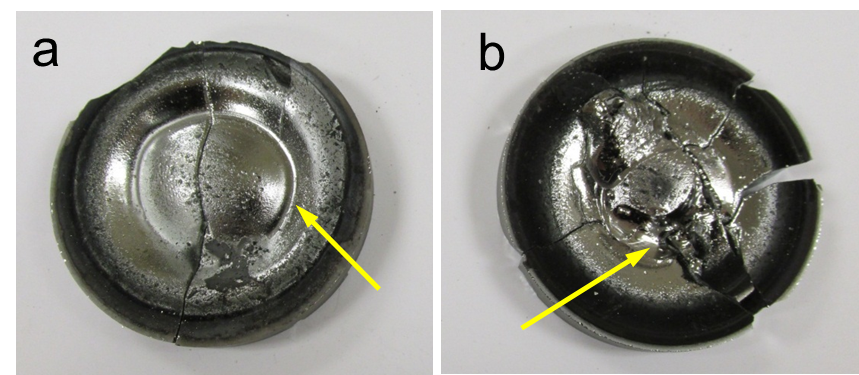
**

**Figure 2. Target material differences between the conventional magnetron sputtering and the molten target sputtering.** Photo of the sputtered solid target with a trace (indicated by arrow) after the conventional magnetron deposition (a). Photo of the molten and solidified target with plasma trace and irregular shape (indicated by arrow) after the molten target sputtering process (b). The plasma trace is because of a recycled target after the conventional sputtering process.

**Supporting information 4. Investigation of the sputtering targets after process**

The MTS demonstrates the molten state of Ge starting from the solid-state target which has 938^o^C melting point under 7mTorr working pressure.


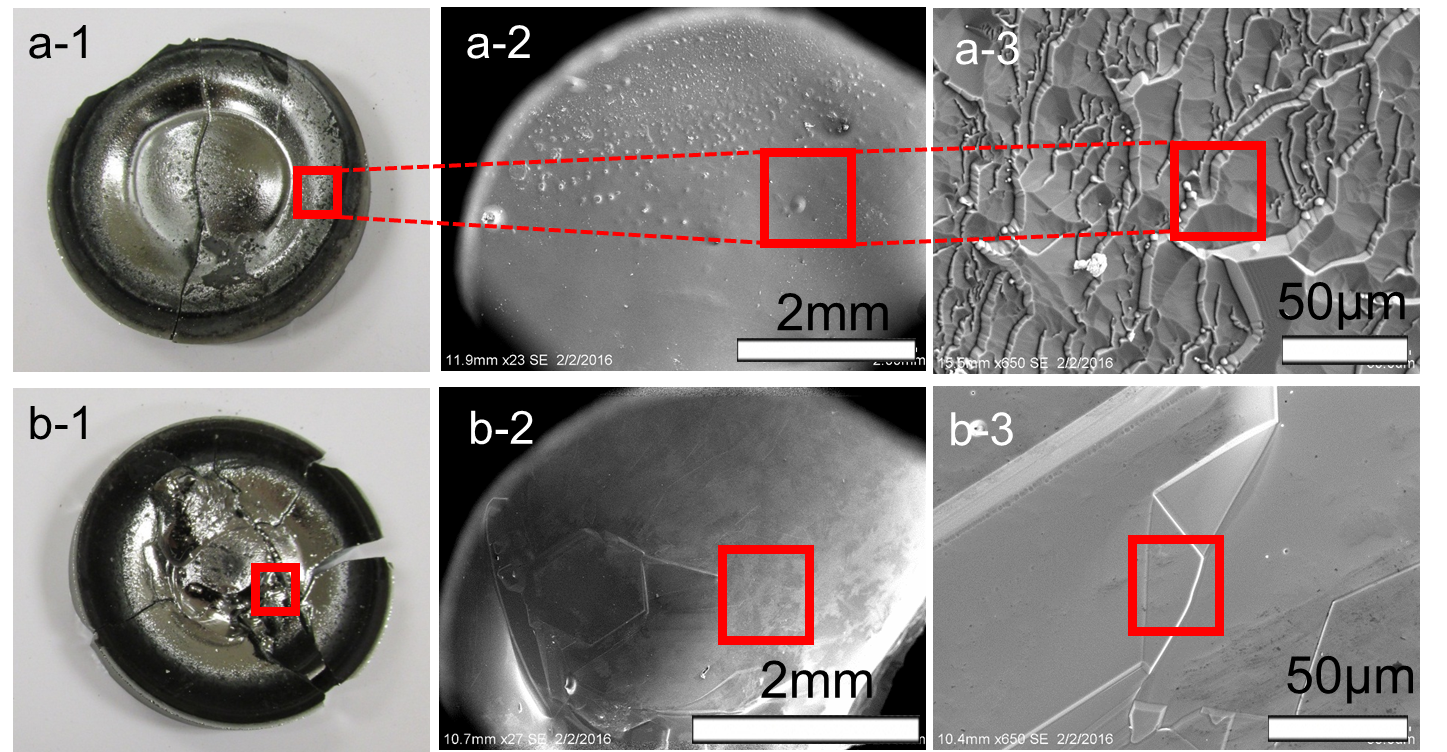


**Figure 3. Photo and SEM images of the Ge target after the convention magnetron sputtering from cold (solid-state) target (a) and MTS with molten target (b).** Photo of the circular groove on the Ge target from the plasma after the conventional sputtering (a-1). Photo of an uneven shape of Ge target from the MTS (b-1). The surface of the solid target from the conventional magnetron sputtering is rough and has steps like figure (a-2 and a-3). On the other hand, figure (b-2 and b-3) shows the molten target surface is smooth.

**Supporting information 5. Sputtering target TEM study**

The MTS demonstrates molten-state (liquid-phase) of Ge from the modified magnetron gun by removing the water cooling channel by the ring groove below the target for the conductive heat sink attached to the conventional magnetron sputtering system.

**
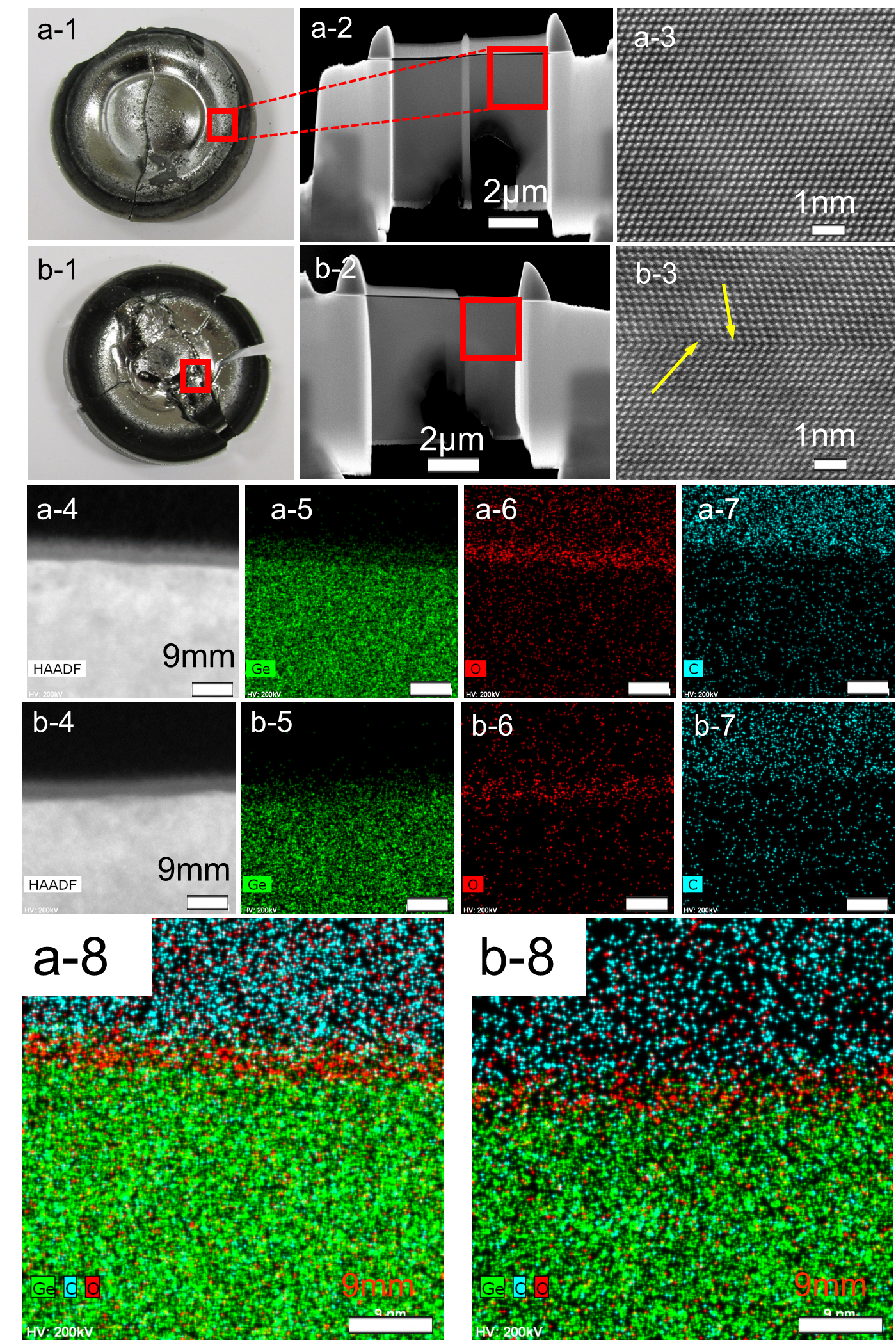
**

**Figure 4. High-resolution TEM (HRTEM) images and EDX mapping of Ge targets after sputtering by using (a) the conventional system and (b) the molten target system (MTS).** The photo and TEM images of the solid Ge target from a conventional sputtering system (a-1 and a-2) and the photo and TEM images of the Ge target from the MTS (b-1 and b-2). The TEM samples were prepared by focused ion beam (FIB) lithography. The HRTEM image of the Ge targets from the conventional sputtering (a-3) and MTS (b-3), respectively. The twin lattice and stacking faults in the Ge target and supports that the Ge target was melted and then solidified (b-3). HRTEM HAADF images of the Ge targets from the conventional and MTS systems are shown (a-4 and b-4), respectively. Energy Dispersive X-ray (EDX) element mapping of Ge (a-5 and b-5), O (a-6 and b-6), C (a-7 and b-7) and combined Ge / C / O (a-8 and b-8). The composition ratio is the same between the Ge targets from both conventional sputtering and the molten target sputtering. Both have oxide layers on the Ge target surface.
